# Acute Liver Failure With Transient Liver Steatosis Following Multiple Hits Postoperatively in a Patient With Limb‐Girdle Muscular Dystrophy: A Case Report

**DOI:** 10.1002/ccr3.72009

**Published:** 2026-02-06

**Authors:** Anders Benjamin Kildal, Espen Molden, Elisabeth Myrseth, Didrik Kjønås, Gunnar Oltmanns, Rasmus Goll, Kim Erlend Mortensen, Geir Ivar Nedredal

**Affiliations:** ^1^ Anesthesia and Critical Care Research Group, Department of Clinical Medicine, Faculty of Health Sciences UIT—The Arctic University of Norway Tromsø Norway; ^2^ Department of Anesthesiology and Intensive Care University Hospital of North Norway Tromsø Norway; ^3^ Center for Psychopharmacology, Pharmacogenetics Unit Diakonhjemmet Hospital Oslo Norway; ^4^ Department of Digestive Surgery University Hospital of North Norway Tromsø Norway; ^5^ Department of Radiology University Hospital of North Norway Tromsø Norway; ^6^ Department of Medical Gastroenterology University Hospital of North Norway Tromsø Norway; ^7^ Gastroenterology and Nutrition Research Group, Department of Clinical Medicine, Faculty of Health Sciences UIT—The Arctic University of Norway Tromsø Norway

**Keywords:** acetaminophen, computed tomography, CYP1A2, CYP2D6, CYP3A4, fatty liver, neuromuscular disease, paracetamol, UGT2B15, ultrasound

## Abstract

Transient liver steatosis is rarely described. A fast development of liver steatosis leading to acute liver failure (ALF) is, to our knowledge, rarely observed. It is so far observed and published in acute fatty liver of pregnancy, and in a few cases of ALF. However, it is observed in elective surgery for pancreaticoduodenectomy and some cases of cytostatic treatment, without subsequent development of ALF. Paracetamol toxicity within the maximum daily allowed dosage (and only a few days of paracetamol administration) prior to the development of ALF has been described in eight patients with neuromuscular disease (NMD). These patients carried genotypes consistent with altered drug metabolism, possibly changing the hepatic disposition of paracetamol. In this report, we describe a patient case of limb‐girdle muscular dystrophy, a subgroup of the NMD, that developed acute liver steatosis within 30 h and subsequently ALF. A 36‐year‐old white woman was electively admitted for a surgical diversion with an end‐colostomy due to chronic constipation. Postoperatively, she was exposed to different factors potentially affecting liver function (multiple hits against the liver), such as paracetamol administration in maximal daily allowed dose, a hypotensive event, and a redo surgery due to perforated colon on postoperative Day 21. Subsequently, she developed severe ALF three days later. The patient responded to standard medical treatment for ALF and was discharged 2 months after the initial hospital admission. Pharmacogenetic analyses indicated a change in paracetamol metabolism towards increased level of toxic metabolite. Six months after the admission, both CT and MR scans showed complete regression of the liver steatosis; this was in addition confirmed with normal liver elastography. To our knowledge, this is the first reported clinical observation of a transient acute liver steatosis with complete regression to normal liver function and morphology. Moreover, it is of great importance to early recognize the development of acute steatosis since it is one of multiple liver hits that predisposes these patients to develop ALF. The importance of pharmacogenetics for risk in paracetamol‐induced liver toxicity should be further investigated.

## Key Clinical Message

Acute liver failure may occur in limb‐girdle muscular dystrophy despite paracetamol use within recommended limits, particularly when multiple hepatic hits are present. Clinicians should consider pharmacogenetic variability and maintain awareness of transient hepatic steatosis on CT or MR imaging, as its presence may predispose patients to acute liver failure.

**Abbreviations**
ALF: Acute liver failure
ASH: Alcoholic steatohepatitis
BID: Twice a day
CT: Computed tomography
CYP: Cytochrome P450
HU: Hounsfield Units
ICU: Intensive care unit
L‐S: Liver‐minus‐spleen
MELD: Model for End‐Stage Liver Disease
MRI: Magnetic resonance imaging
NAFLD: Non‐alcoholic fatty‐liver disease
NAPQI: N‐acetyl‐p‐benzoquinone imine
NASH: Non‐alcoholic steatohepatitis
NMD: Neuromuscular Disease
QID: Four times a day
TID: Three times a day
US: Ultrasound

## 1 Introduction

Hepatic steatosis has a prevalence of 25% worldwide [1]. To our knowledge, the only clinically radiological observed sequence from a normal to a fatty liver, and then recovery back to normal liver, has been reported in patients who underwent elective pancreaticoduodenectomy [2], in patients treated with the combination of interferon alfa‐2A and 5‐fluorouracil for metastatic colorectal carcinoma [3] and in patients with breast cancer treated with tamoxifen (a selective estrogen receptor modulator) [4]. The change from normal to fatty liver was detected within a minimum of 1 month in the two aforementioned studies with pancreaticoduodenectomy [2] and treatment with interferon alfa‐2A and 5‐fluorouracil [3]. This coincided with the start of treatment to demonstration of fatty liver. However, in an acute clinical setting, it has been observed the sequence from fatty to normal liver in acute fatty liver of pregnancy [5] and some acute liver failure (ALF) cases [6]. Experimental studies have shown that fatty liver can develop quickly [7] and likewise resolve quickly [8]. However, the paucity in the literature of a quick appearance and disappearance of fatty liver in the Intensive Care Unit (ICU) setting is either due to underreporting or lack of diagnostic workup. Moreover, this entity may not be observed, especially when a complication such as ALF is not developed requiring a mandatory diagnostic workup.

Eight cases of paracetamol toxicity in patients with neuromuscular disease (NMD) with no prior known liver disease have been described in five papers [9, 10, 11, 12, 13] all related to postoperative pain medications or critical illness. The common denominators for these patients were paracetamol use below or up to the maximum recommended daily dosage and short duration of paracetamol administration combined with one or multiple other hits until the development of ALF. These studies have been extensively summarized in the paper by Lao et al. [12]. Only one of the reported NMD cases was a patient with limb‐girdle muscular dystrophy [13].

In the case report by Lao et al. [12] measured genotypes of cytochrome P450 (CYP) enzymes were discussed to imply risk of increased level of the toxic paracetamol metabolite, N‐acetyl‐p‐benzoquinone imine (NAPQI). Paracetamol is subjected to metabolism via several phase I and phase II enzymes exhibiting genetic polymorphism, including CYP1A2, CYP2D6, CYP3A4, and UGT2B15 [14]. The pharmacogenetic profile of the patient presented in the case report by Lao et al. [12] suggested increased activities of CYP1A2 and CYP3A4, while CYP2D6 and UGT1B15 activities were decreased. The combination of the genotypes was interpreted to imply higher formation of NAPQI via CYP1A2 and CYP3A4, increased availability of paracetamol for CYP1A2 and CYP3A4 metabolism due to inactive CYP2D6 metabolism, and then finally reduced trapping of NAPQI due to UGT1B15 glucuronidation.

Based on the case report of Lao et al., pharmacogenetic analyses were included in the present report to highlight the potential importance of genetic differences in drug‐metabolizing enzymes for the susceptibility of paracetamol‐induced liver toxicity. Moreover, this case report emphasizes the relevance of slower drug metabolism in patients with NMD. Furthermore, it is discussed whether the multiple hit hypothesis [15, 16] is also applicable to describe the pathophysiology of ALF following acute steatosis.

## 2 Case History

**FIGURE 1:**
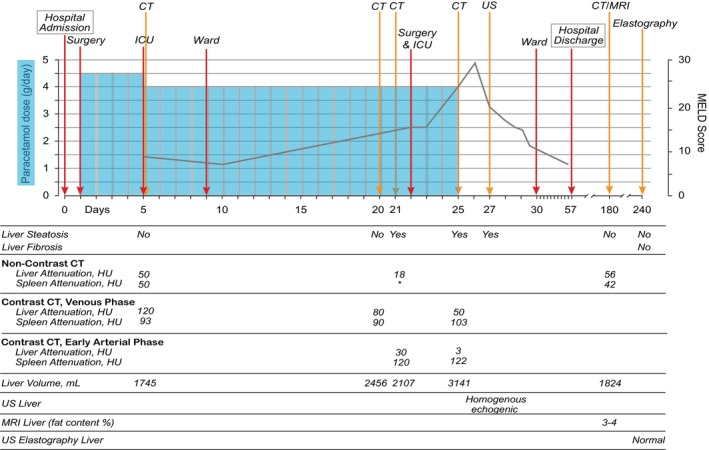
Timeline presenting time points for liver imaging, hospital admission and discharge, admission to Intensive care Unit (ICU) and ward, and surgery. Additionally, there is also presented the evolution of paracetamol dosage and Model for End‐Stage Liver Disease (MELD) score [17] in the observation period. The MELD score is based on laboratory parameters and has a range from 6 to 40, in which a higher score predicts a higher 3‐month mortality related to liver disease. As defined earlier by Wells et al. [18], early arterial phase contrast computed tomography (CT) scans were considered as a non‐contrast CT for the purposes of steatosis analysis. CT criteria for steatosis in non‐contrast CT and CT with contrast in the early arterial phase included liver attenuation 10 Hounsfield Units (HU) less than spleen attenuation and absolute liver attenuation of less than 40 HU. CT criterion for steatosis on CT with contrast in the venous phase was defined as liver attenuation of less than 20 HU than spleen attenuation. * Imaging the spleen was not included in this specific non‐contrast CT scan. Other abbreviations: Magnetic resonance imaging (MRI) and ultrasound (US).

A 36‐year‐old woman with limb‐girdle muscular dystrophy in need of a wheelchair, respiratory failure secondary to her NMD requiring nocturnal BiPAP (bilevel Positive Airway Pressure), and daily use of a cough assist machine. She had, for a long time, struggled with significant defecation and obstipation problems. Therefore, she was scheduled for elective surgery for diversion with an end‐colostomy. For postoperative analgesia, paracetamol was administered 1.5‐g TID (i.e., 4.5 g per day) from Day 1 until Day 5, and then changed from Day 5 to 1‐g QID (i.e., 4 g per day; See Figure 1) for timeline including paracetamol usage and progress of model of end‐stage liver disease (MELD) score [17]. Her weight and height were 95 kg and 163 cm, respectively, corresponding to an ideal body weight of 55 kg and a BMI of 35.8 kg/m^2^. At Day 5, she was admitted to the ICU due to paralytic colon ileus, abdominal pain and respiratory failure. On Day 21 she developed a severe hypotensive bleeding which responded to blood transfusions. The next day she had emergency surgery due to a perforated colon with abdominal contamination. Additionally, piperacillin/tazobactam 4‐g/0.5‐g TID was initiated. On Day 25 she developed severe ALF with excessive vasoplegia which required high dose vasopressors, as represented by noradrenaline infusion of > 800 ng/kg/min and moreover vasopressin infusion of 0.04 IU/min. Paracetamol was stopped on the morning of Day 25.

## 3 Differential Diagnosis, Investigations, and Treatment

Based on biochemical lab tests, indicating ALF, standard medical therapy was initiated with intravenous N‐acetylcysteine regimen (150 mg/kg over 1 h, 50 mg/kg over 4 h, 100 mg/kg over 16 h), an anidulafungin loading dose of 200 mg, thiamin 100 mg BID, and hydrocortisone 50 mg QID. Additionally, one fytomenadion dose of 10 mg, 300 mL of 20% albumin and 400 mL of fresh frozen plasma was given. The next day (Day 26) vasopressin infusion was stopped, and noradrenaline infusion was subsequently reduced. Serum paracetamol was increased (179 umol/L; 10.5 h after last dose of paracetamol) at Day 25 and reduced to 65 and 55 umol/L 24 and 26 h after last dose of paracetamol, respectively. The elimination half‐life of paracetamol based on these serum measurements in this critical setting was estimated to be 10–11 h (5× more than normal elimination half‐life of paracetamol). The patient was discharged from the ICU to the surgical ward at Day 30, and later discharged from the hospital 2 months after hospital admission. Of note, 10 years prior to this hospital admission, the patient was troubled with chronic back pain with extensive daily use of 1‐g QID of paracetamol for a year, with no signs of liver failure.

**FIGURE 2:**
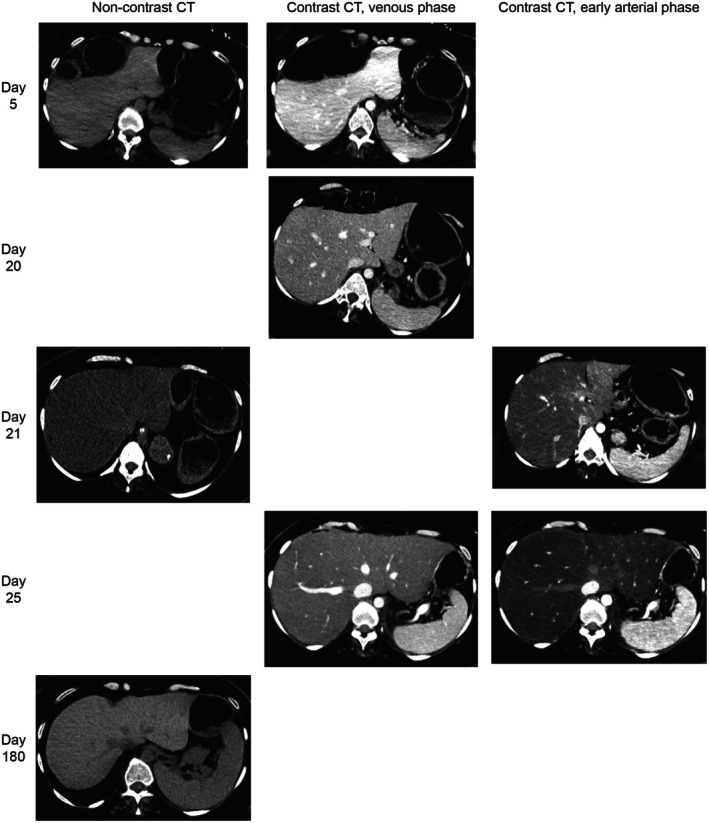
All computed tomography (CT) scans taken throughout the observation period. All CT scans are presented in axial reconstructions with Window Width of 170 Hounsfield Units (HU) and Window level 70 HU. All early arterial phase contrast CT scans in this report were CT angiography protocols (≤ 20 s delay after contrast injection) and all venous phase contrast CT were taken with an approximately 70 s' delay after contrast injection. The respective liver and spleen attenuations numbers from each CT scan are presented in Figure 1.

**FIGURE 3:**
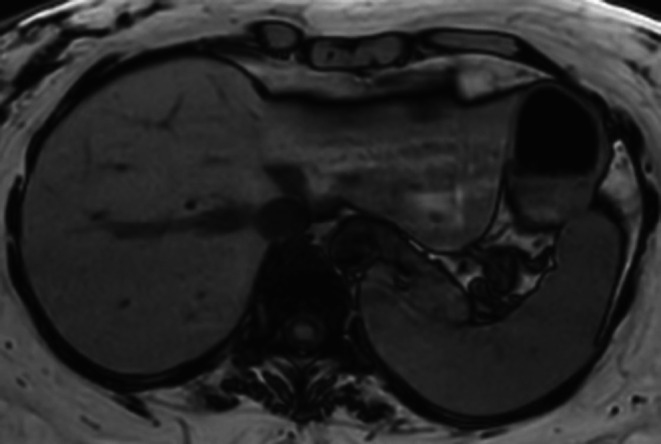
Magnetic resonance imaging (MRI; T1‐weighted duo‐echo out‐of‐phase) presented in an axial plane reconstruction from day 180 after hospital admission. The protocol also included Dixon fat quantifying sequences estimating hepatic fat content to be 3%–4%.

At day 20, a computed tomography (CT) scan showed a normal liver, while the repeated scan the following day revealed acute steatosis (Figures 1 and 2). The scan on Day 25 and liver ultrasound on Day 27 showed persisting steatosis. There were no signs of congestive hepatopathy or periportal edema. Six months after the hospital admission, both CT and magnetic resonance imaging (MRI; Figure 3) scans of the liver showed complete recovery of the steatosis observed first time on Day 21. The complete recovery was also confirmed with a FibroScan (4,0 kPa) of the liver six months after the hospital discharge.

## 4 Conclusions and Results

After the clinical course, a blood sample from the patient was shipped for pharmacogenetic analysis at an accredited laboratory. Strikingly, the pharmacogenetic profile of the current patient was very similar to the patient described by Lao et al. [12]. The analyses showed that our patient was carrying the following genotypes of the tested drug‐metabolizing enzymes: CYP2D6*3/*10, CYP1A2*1/*1F, CYP3A4*1/*1, CYP3A5*3*3 and UGT2B15*1/*2. These genotypes are associated with strongly reduced CYP2D6 activity, increased CYP1A2 activity, and reduced UGT2B15 activity, while genotypes of CYP3A4/3A5 indicate normal enzyme activities. Thus, the pharmacogenetic profile of our patient was very similar to that found for a previous reported patient with NMD [12]. The strongly reduced CYP2D6 metabolism, which implies reduced activity of the metabolic pathway producing the non‐toxic metabolite, may accordingly shift the conversion of paracetamol to the cytotoxic metabolite NAPQI via the increased CYP1A2 activity as observed in our patient. Lastly, the reduced UGT2B15‐mediated glucuronidation in our patient possibly reduced trapping (deactivation) of NAPQI. Furthermore, glutathione synthesis depends on glutamine from the skeletal muscle to form glutamate [19]. Thus, patients with limb‐girdle muscular dystrophy may have lower concentration of glutathione compared to healthy due to their altered body composition with low skeletal muscle mass. Finally, the interplay between the respective pharmacogenotypes may explain why our patient was predisposed for an increased risk of paracetamol‐induced liver toxicity, albeit in synergy with other hits against the liver.

## 5 Discussion

This case report describes ALF with acute steatosis following prolonged postoperative paracetamol administration in therapeutic doses in combination with multiple liver hits such as a prolonged hypotensive event, septicemia, and acute surgery. No other liver toxic drugs were found during retrospective audit of the electronic journal. Additionally, her limb‐girdle muscular dystrophy and pharmacogenetic profile implying increased hepatic level of NAPQI probably made her more susceptible for developing a drug‐induced liver injury.

To our knowledge this is the first clinical observation of serial CT scans with a normal hepatic CT scan 30 h prior to the next CT scan revealing acute hepatic steatosis. In experimental animal studies, 24 h is to our knowledge the fastest radiological observed change from normal to fatty liver. This was presented in a study in which rats were given L‐ethionine intraperitoneally [7], in which a non‐contrast CT was taken at baseline and repeated in the same animals 24 h after given L‐ethionine. Furthermore, the fastest radiological change in humans from normal to fatty liver was based on our literature search to be observed by abdominal ultrasound before and after 1 week of binge‐drinking at Roskilde festival, in which 4 of 14 study participants developed hepatic steatosis [20].

A case report of a patient who underwent pancreaticoduodenectomy due to cancer, the preoperative CT revealed normal liver. However, a CT scan 3 months postoperatively revealed steatosis. The liver recovered partly after two months of treatment with zinc and pancreatic enzyme supplementation [21]. Moreover, in a retrospective study of 60 nonalcoholic patients after pancreaticoduodenectomy with no prior steatosis, 14 of the patients developed steatosis in a follow‐up CT scan after 4–12 months. The steatosis was partially to fully reversed after the initiation of 12‐months of pancreatic enzyme supplementation [22]. Tang et al. reported in 17 patients with acute fatty liver of pregnancy the reversal from hepatic steatosis at admission to complete normal liver within 4 weeks after treatment with plasma exchange and continuous renal replacement [5]. In a retrospective study of 177 patients with ALF, CT scans of 103 patients were available and only 22 patients had follow‐up CT scans. Hepatic steatosis was present in 74% of the patients. In 4 out of 7 patients who recovered with medical treatment, the hepatic steatosis recovered to normal liver, and none had initial steatosis on the second scan. None of the patients with multiple scans (*n* = 15) who either died or had liver transplantation had a change from hepatic steatosis to normal liver on the second scan [6]. From this we can conclude that patients with fatty liver development who are reversible with medical treatment are not susceptible to liver failure.

Three and two out of 8 CT scans in this case report were venous and early arterial contrast enhanced CT scans, respectively. In non‐contrast CT scans, moderate to severe steatosis (minimum 30% fat content analyzed in liver biopsies) is predicted by relative hypoattenuation in two different fashions [18]: (1) A liver‐minus‐spleen (L‐S) difference attenuation lower than minus 10 Hounsfield units (HU; [23]), and (2) low absolute attenuation with liver attenuation lower than 40 HU [24]. However, in venous contrast enhanced CT scans, moderate to severe steatosis detected in liver biopsies has been shown to be predicted by an L‐S difference attenuation lower than minus 19 HU [25]. Also including comparison to non‐contrast CT (with the abovementioned threshold for diagnosing hepatic steatosis), the threshold of detection of moderate to severe steatosis with L‐S difference attenuation in venous contrast enhanced CT can be further discussed. Studies have shown L‐S difference attenuation ranging from less than minus 20 HU to less than minus 43 HU in the portal venous phase. The range depends accordingly on the injection protocol [26, 27].

The multiple hit hypothesis [15, 16] has been used to describe how chronic alcohol usage or obesity, and diabetes over time leads to steatosis with subsequent development of alcoholic and non‐alcoholic steatohepatitis (ASH and NASH, respectively) and further to end‐stage liver failure with the development of cirrhosis. This case report elaborates on whether the multiple hit hypothesis can be applicable to describe the pathophysiology of the development of ALF following acute steatosis.

The multiple‐hit hypothesis seems applicable in this patient, as several risk factors likely acted in synergy to predispose her to ALF despite paracetamol exposure within the therapeutic range for otherwise healthy individuals. (i) Her limb‐girdle muscular dystrophy was associated with increased body fat percentage, low skeletal muscle mass, low physical activity, and high sedentary behaviour, all of which are independently linked to NAFLD [28, 29]. (ii) Her pharmacogenetic profile for paracetamol metabolism may have predisposed her to an imbalance between detoxification pathways and the generation of hepatotoxic metabolites. (iii) She received paracetamol continuously for 21 days prior to a hypotensive bleeding episode and emergency surgery. (iv) Additional systemic insults such as the hypotensive event, major abdominal surgery, and subsequent critical illness likely increased hepatic vulnerability through ischemia, inflammation, and impaired glutathione homeostasis. In line with previous reports of paracetamol toxicity in NMD patients, the pattern is consistent: dosages remained within the recommended range for otherwise healthy individuals, yet the presence of one or more additional insults preceded the development of ALF.

Reduced glutathione levels in muscle have been shown in critical illness [30]. Due to her low skeletal muscle mass, her glutathione stores may have been more easily depleted compared to other patients. Such depletion may have heightened her susceptibility to paracetamol‐induced hepatotoxicity, even when administered at therapeutic doses [31, 32]. She received 42–47 mg/kg/day or 72–81 mg/kg/day of paracetamol, depending on whether the calculation was based on her actual or ideal body weight, respectively. This contrasts with the FDA recommendation of a maximum of 15 mg/kg per dose (up to 4000 mg/day), which corresponds to approximately 60 mg/kg/day for a 66 kg adult. Moreover, a pharmacokinetic study in healthy adults and children with spinal muscular atrophy (SMA) demonstrated reduced clearance and slower metabolism of paracetamol, with one of 12 SMA patients developing liver involvement [33]. Despite these findings, the authors recommended a dose of 15 mg/kg per dose (maximum 4 g/day) in SMA adults, accompanied by monitoring of standard liver biomarkers 48 h after the initiation of paracetamol treatment.

To our knowledge, this is the first clinical observation of transient acute steatosis with complete regression to normal liver. Accordingly, the existence of hepatic steatosis in the acute setting of a disease is a risk factor the clinician should be aware of, since steatosis is one of the multiple liver hits that can predispose these patients to the severe condition of ALF. In patients with limb‐girdle muscular dystrophy, paracetamol should be limited to ≤ 15 mg/kg per dose (calculated using the lowest of ideal or actual body weight), with a maximum of 4000 mg/day, and used with caution during prolonged administration, particularly in the setting of critical illness, which is often associated with multiple physiological stressors. Pharmacogenetic variability in drug‐metabolizing enzymes should be studied further as predisposing for paracetamol‐induced liver toxicity within recommended doses. Furthermore, additional studies are warranted to investigate paracetamol pharmacokinetics in individuals with low skeletal muscle mass and NMD, as well as in the critically ill setting.

## Author Contributions

**Anders Benjamin Kildal:** conceptualization, investigation, supervision, validation, visualization, writing – original draft, writing – review and editing. **Espen Molden:** conceptualization, investigation, methodology, validation, visualization, writing – review and editing. **Elisabeth Myrseth:** conceptualization, writing – review and editing. **Didrik Kjønås:** conceptualization, writing – review and editing. **Gunnar Oltmanns:** conceptualization, investigation, methodology, validation, visualization, writing – review and editing. **Rasmus Goll:** conceptualization, investigation, methodology, validation, writing – review and editing. **Kim Erlend Mortensen:** conceptualization, validation, writing – review and editing. **Geir Ivar Nedredal:** conceptualization, investigation, supervision, validation, visualization, writing – review and editing.

## Funding

The authors have no funding sources to declare.

## Ethics Statement

The authors have nothing to report.

## Consent

Written informed consent was obtained from the patient for publication of this case report and any accompanying images. A copy of the written consent is available for review by the Editor‐in‐Chief of this journal.

## Conflicts of Interest

The authors declare no conflicts of interest.

## Data Availability

The authors have nothing to report.
